# Supplementary material for: Investigation of Anticonvulsant Potential of Morus alba, Angelica archangelica, Valeriana officinalis, and Passiflora incarnata Extracts: In Vivo and In Silico Studies
Source: Int J Mol Sci. 2025 Jul 3;26(13):6426. doi: 10.3390/ijms26136426 (PMC12250342; doi:10.3390/ijms26136426)
Supplement: Supplementary file 1 [file ijms-26-06426-s001.zip › ijms-3697600-supplementary.pdf]

International Journal of
Molecular Sciences

MDPI

Supplementary Materials: Investigation of Anticonvulsant Potential of *Morus alba, Angelica archangelica, Valeriana officinalis,* and *Passiflora incarnata* Extracts: In Vivo and In Silico Studies

Felicia Suciu, Dragos Paul Mihai *, Anca Ungurianu, Corina Andrei, Ciprian Pușcașu, Carmen Lidia Chițescu, Robert Viorel Ancuceanu, Cerasela Elena Gird, Emil Stefanescu, Nicoleta Blebea, Violeta Popovici *, Adrian Cosmin Rosca, Cristina Isabel Viorica Ghiță and Simona Negres

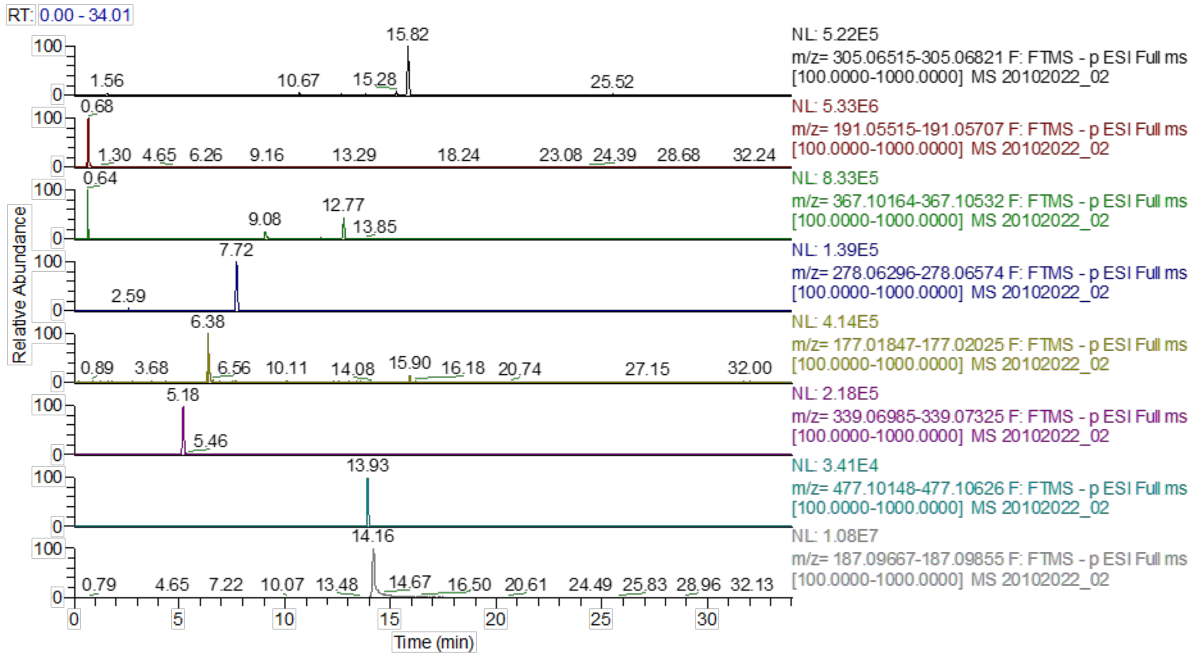

Figure S1. UHPLC-HRMS/MS chromatogram of *Angelica archangelica* extract, in which the following compounds were identified (from top to bottom): gallocatechin (m/z: 305.06668, Rt: 21.17), quinic acid (m/z: 191.05611, Rt: 0.68), feruloylquinic acid (m/z: 367.10164, Rt: 12.77), aesculetin (m/z: 177.01936, Rt: 6.38), daphnin (m/z: 339.07215, Rt: 5.18), isorhamnetin-3-O-hexoside (m/z: 477.10387, Rt: 13.93), and azelaic acid (m/z: 187.09761, Rt: 14.16).

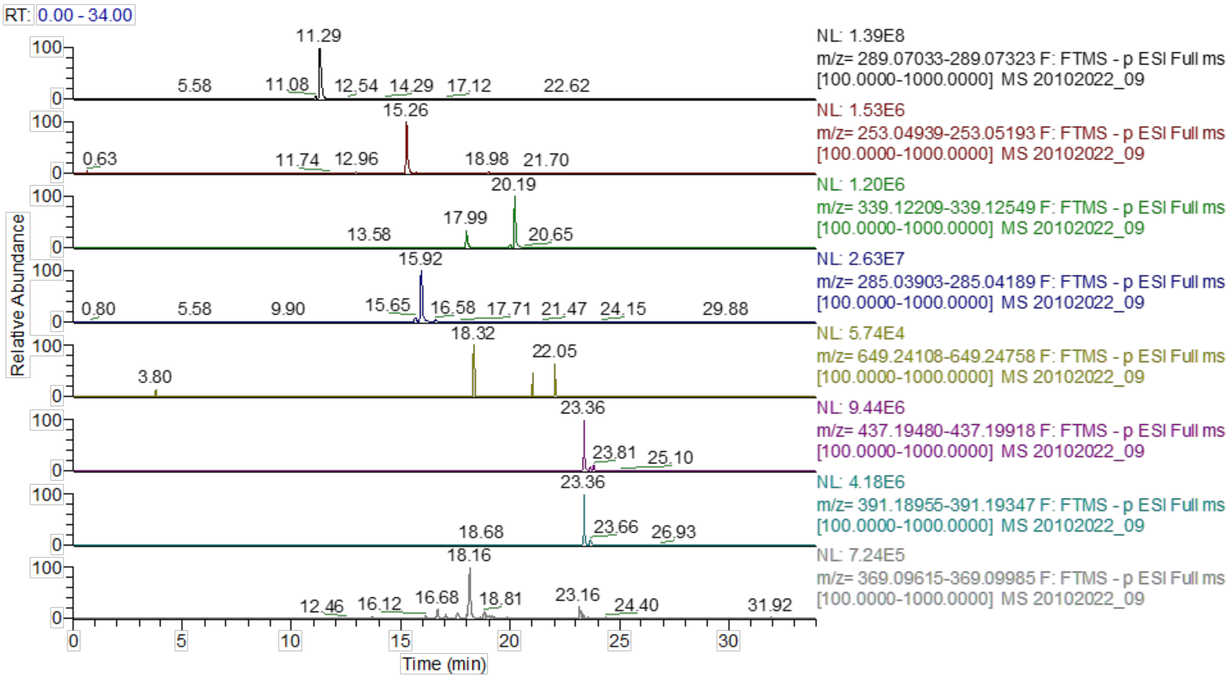

Figure S2. UHPLC-HRMS/MS chromatogram of *Morus alba* extract, in which the following compounds were identified (from top to bottom): catechin (m/z: 289.07176, Rt: 11.29), daidzein (m/z: 253.05066, Rt: 15.26), baptigenin (m/z: 285.04046, Rt: 15.92), 2′-hydroxygenistein (m/z: 649.24108, Rt: 18.32), alopecurone A (m/z: 311.04085, Rt: 9.98), lechianoine A (m/z: 311.04085, Rt: 9.98), prostatrol F (m/z: 311.04085, Rt: 9.98), and sifroronol A (m/z: 311.04085, Rt: 9.98).

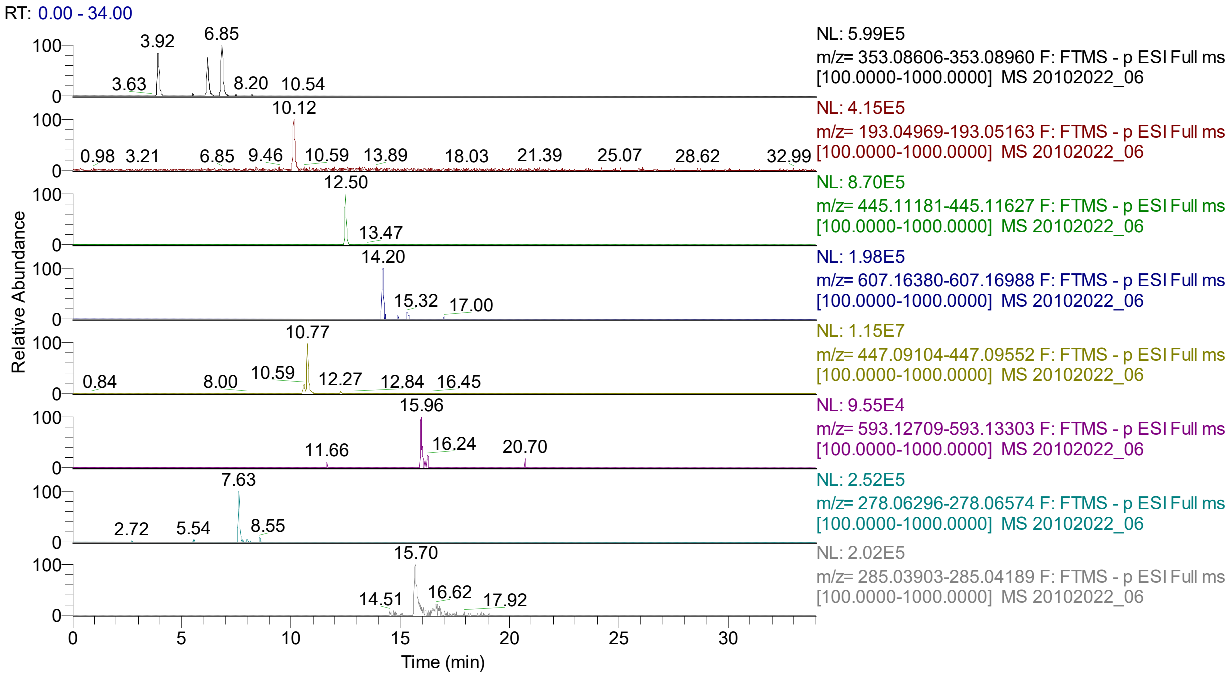

Figure S3. UHPLC-HRMS/MS chromatogram of *Passiflora incarnata* extract, in which the following compounds were identified (from top to bottom): neochlorogenic acid (m/z: 353.08783, Rt: 3.92), chlorogenic acid (m/z: 353.08783, Rt: 6.85), biochanin A glucoside (m/z: 445.11404, Rt: 12.50), diosmetin 7-O-rutinoside (m/z: 607.16684, Rt: 14.20), kaempferol-O-glucoside (m/z: 447.09331, Rt: 10.77), procyanidin (m/z: 593.13006, Rt: 15.96), caffeoylshinic acid (m/z: 278.06435, Rt: 7.63), and baptigenin (m/z: 285.04046, Rt: 15.70).

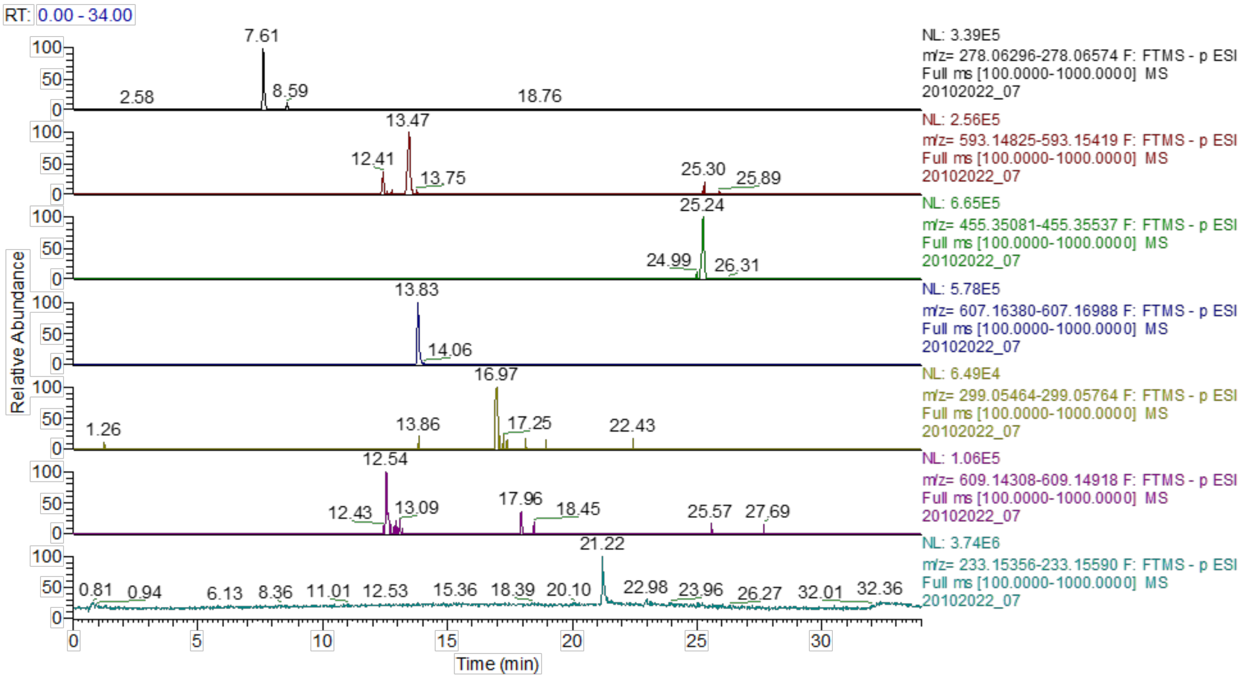

Figure S4. UHPLC-HRMS/MS chromatogram of *Valeriana officinalis* extract, in which the following compounds were identified (from top to bottom): caffeoylshinic acid (m/z: 278.06435, Rt: 7.61), apigenin 7-O-glucosylglucoside (m/z: 593.15122, Rt: 13.47), oleanolic acid (m/z: 455.35309, Rt: 25.24), hispidulin 7-rutinoside (m/z: 607.16684, Rt: 13.83), hispidulin (m/z: 299.05614, Rt: 16.97), rutin (m/z: 609.14613, Rt: 12.54), and valerenic acid (m/z: 233.15473, Rt: 21.22).
